# Cerium-Doped Self-Assembling Nanoparticles as a Novel Anti-Oxidant Delivery System Preserving Mitochondrial Function in Cortical Neurons Exposed to Ischemia-like Conditions

**DOI:** 10.3390/antiox12020358

**Published:** 2023-02-02

**Authors:** Valeria Nele, Valentina Tedeschi, Virginia Campani, Raffaella Ciancio, Alessia Angelillo, Sossio Fabio Graziano, Giuseppe De Rosa, Agnese Secondo

**Affiliations:** 1 Department of Pharmacy, University of Naples Federico II, Via D. Montesano, 80131 Naples, Italy; 2 Division of Pharmacology, Department of Neuroscience, Reproductive and Odontostomatological Sciences, School of Medicine, University of Naples Federico II, Via S. Pansini 5, 80131 Naples, Italy

**Keywords:** lipid nanoparticles, ROS-scavenging, mitochondrial disfunction, cerium oxide, neurons, hypoxia

## Abstract

Neurodegenerative diseases are characterized by mitochondrial dysfunction leading to abnormal levels of reactive oxygen species (ROS), making the use of ROS-scavenging nanomaterials a promising therapeutic approach. Here, we combined the unique ROS-scavenging properties of cerium-based nanomaterials with the lipid self-assembling nanoparticles (SANP) technology. We optimized the preparation of cerium-doped SANP (Ce-SANP) and characterized the formulations in terms of both physiochemical and biological properties. Ce-SANP exhibited good colloidal properties and were able to mimic the activity of two ROS-scavenging enzymes, namely peroxidase and super oxide dismutase. Under ischemia-like conditions, Ce-SANP could rescue neuronal cells from mitochondrial suffering by reducing ROS production and preventing ATP level reduction. Furthermore, Ce-SANP prevented mitochondrial Ca^2+^ homeostasis dysfunction, partially restoring mitochondrial Ca^2+^ handling. Taken together, these results highlight the potential of the anti-oxidant Ce-SANP platform technology to manage ROS levels and mitochondrial function for the treatment of neurodegenerative diseases.

## 1. Introduction

Neurodegenerative diseases induce a progressive loss of function and degeneration of neurons which ultimately leads to death. Common neurodegenerative diseases include amyotrophic lateral sclerosis, Alzheimer’s disease, Parkinson’s disease, and Huntington’s disease [1]. A conspicuous neurodegeneration is also present in stroke, a neurological event caused by a compromised blood supply to the brain which leads to oxygen and glucose deficiencies in specific brain areas. Current therapeutic options for stroke treatment are limited to clot removal by alteplase, moderate hypothermia and mechanical thrombectomy [2]. Therefore, the development of new treatment options for ischemic stroke is an important need of modern medicine. In general, the number of patients affected by this specific neurological event and other neurodegenerative diseases is expected to increase in the next decades due to an increase in the aging population, which poses a serious economic burden on the healthcare system [3]. For example, while nowadays ~15 million people suffer from Alzheimer’s disease worldwide, it is anticipated that this number will grow to 20 million cases in Europe and in the United States by 2050 [4]. There is currently no cure for neurodegenerative diseases and the available treatments are only palliative, which makes the development of effective therapeutics of paramount importance.

Oxidative stress is ubiquitous across neurological diseases and plays a key role in their development [5]. It is typically triggered by an abnormal accumulation of reactive oxygen species (ROS) such as super-oxide anions (O_2_^•−^), hydrogen peroxide (H_2_O_2_), and hydroxyl radicals (HO^•^) which can induce severe cellular damage [6]. Neuronal cells are highly susceptible to ROS due to the presence of high concentrations of redox active metals and oxidation-sensitive polyunsaturated fatty acids, as well as to the relative low expression of anti-oxidant enzymes [7]. This has prompted the development of therapeutic approaches based on the use of inorganic nanomaterials with ROS-scavenging properties [8]. In this context, cerium-based nanoparticles are particularly interesting since the surface cerium atoms can undergo oxidation from Ce^3+^ to Ce^4+^ in the presence of ROS, mimicking the activity of ROS-scavenging enzymes, such as super oxide dismutase (SOD) and peroxidase [9].

A wide variety of cerium-based nanomaterials has been developed over the years for applications in drug delivery and biosensing. Example includes cerium oxide nanoparticles, which have been synthesized in various shapes and sizes and have been shown to possess oxidase-, peroxidase-, catalase-, SOD-, phosphatase-, and haloperoxidase-like activity [10]. Cerium phosphate nanoparticles can also exhibit peroxidase-like activity [11] and have enhanced peroxidase- and catalase-like activity when combined with cerium oxide in nanocomposite structures [12]. Cerium fluoride nanoparticles are another class of cerium-based nanomaterials with peroxidase-like activity and the ability to exert an anti-oxidant effect in vitro in the presence of hydrogen peroxide [13]. Given their favorable properties, cerium-based nanoparticles have been successfully proposed for the treatment of various neurodegenerative diseases, including ischemic and hemorrhagic stroke, Parkinson-like disease, Alzheimer’s disease, and amyotrophic lateral sclerosis [14].

Alternative approaches for the treatment of neurodegenerative diseases rely on gene therapy to regulate the expression of target genes or proteins and to correct pathological mutations [15]. Examples include the use of oligonucleotides, such as anti-sense oligonucleotides or small interfering RNA (siRNA), which are able to prevent the production of pathological proteins involved in the progression of neurodegenerative diseases [15,16,17]. However, in vivo delivery of oligonucleotides poses several hurdles associated with poor cellular uptake, off-target effects, and nuclease degradation. This has led to the emergence of vectors for oligonucleotide delivery such as lipid nanoparticles and polymeric nanoparticles [18]. Our group has pioneered the development of hybrid self-assembling nanoparticles (SANP) for the delivery of bisphosphonates [19,20] and nucleic acids [21] to the brain. SANP have an inorganic calcium phosphate (CaP) core enclosed by a lipid shell, whose composition can be tailored to achieve nanoparticle accumulation in specific organs. Lipid SANP formulations exhibit excellent biocompatibility, high nucleic acid encapsulation efficiency and intracellular delivery, and can be prepared by simple component mixing at room temperature without the need of complex manufacturing procedures [21]. The lipid SANP technology has been successfully used to deliver drugs and miRNA or siRNA to the central nervous system to treat brain tumors, while the use of SANP for neurodegenerative diseases is still unexplored.

Given the complex pathology of neurodegenerative diseases, a multi-functional nanoparticle platform with anti-oxidant activity and the ability to deliver specific drugs may be a promising therapeutic strategy. Here, we combined the unique ROS scavenging properties of cerium-based nanomaterials with the lipid SANP technology to produce multi-functional nanoparticles for the treatment of neurodegenerative diseases. We developed a straightforward coprecipitation method to obtain cerium-doped CaP nanoparticles, which were combined with cationic liposomes to yield cerium-SANP (Ce-SANP). The nanoparticles were characterized in terms of colloidal properties, surface charge, stability against aggregation in serum albumin, and enzymatic activity. We then tested their ability to rescue PC12-derived neuronal cells and primary cortical neurons from chemical hypoxia and re-oxygenation (CH/Rx) and oxygen and glucose deprivation followed by reoxygenation (OGD/Rx), two models of brain ischemia which induce oxidative stress. The Ce-SANP exhibited favorable colloidal properties, good stability against aggregation in the presence of serum proteins, as well as peroxidase- and SOD-like activity. They were able to rescue neuronal cells from ROS-induced cell death in a dose-dependent manner and to prevent a reduction in ATP levels and mitochondrial Ca^2+^ homeostasis dysfunction.

## 2. Materials and Methods

### 2.1. Materials

Sodium chloride (NaCl), calcium chloride (CaCl_2_), sodium phosphate dibasic (Na_2_HPO_4_), cerium (III) nitrate, sodium citrate tribasic dihydrate (C_6_H_5_Na_3_O_7_•2H_2_O), ammonia solution (28–30%), and the SOD assay kit (cat. n. 19,160) were obtained from Merck Life Science S.r.l. (Milan, Italy). 1,2-dioleoyl-3-trimethylammonium-propane chloride (DOTAP) was kindly donated by Lipoid GmbH (Ludwigshafen, Germany) while N-palmitoyl-sphingosine-1-{succinyl[methoxy(polyethylene glycol)2000]} (Cer_16_-PEG_2000_) was purchased from Avanti Polar Lipids (Alabaster, USA). The 3,3′,5,5′-Tetramethylbenzidine (TMB) substrate solution (cat. n. N301) and the Stop solution (cat. n. N600) were purchased from ThermoFisher Scientific (Rodano, Italy) while regenerated cellulose (RC) syringe filters with a 0.2 μm pore size were purchased from Exacta + Optech Labcenter SpA (San Prospero, Italy).

### 2.2. Methods

#### 2.2.1. Ce-CaP Nanoparticle Synthesis 

The inorganic cerium-CaP nanoparticles were prepared by a coprecipitation method. Briefly, 1 mL of an aqueous solution containing 30 mM Na_2_HPO_4_ was added dropwise while vortex stirring at 1400 rpm to 1 mL of an aqueous solution containing 25 mM CaCl_2_, 150 mM cerium (III) nitrate, and 100 mM sodium citrate tribasic; 250 μL of 1.4 M NH_4_OH solution was then added dropwise while vortex stirring at 1400 rpm. The obtained nanoparticle suspension was incubated at 37 °C for 5 min, stored at 4 °C overnight, and filtered through a 0.22 μm pore-sized RC membrane. The cerium-CaP nanoparticles were stored at 4 °C until further use. 

#### 2.2.2. Liposome Formulation

Liposomes (DOTAP:Cer_16_-PEG_2000_ 94:6 mol%) were prepared via the thin film hydration method followed by extrusion. The lipids were dissolved in a chloroform:methanol mixture (2:1 *v*/*v*) and transferred in a 50 mL round bottom flask in the appropriate mixing ratios. The organic solvent mixture was removed by rotary evaporation (Laborata 4010 digital, Heidolph, Schwabach, Germany) and the obtained lipid film was hydrated with deionized water for 2 h at 65 °C to a lipid concentration of 2.5 mg/mL. The vesicle suspension was extruded through pore-sized polycarbonate membranes (Nucleopore Track-Etched 25 mm membrane, Whatman, Brentford, UK) with varying pore sizes by using a thermobarrel extruder (Lipex Extruder, Evonik, Essen, Germany) at 65 °C. More specifically, the vesicle suspension was forced through 400 nm membranes (3 passages), 200 nm membranes (3 passages), and 100 nm membranes (5 passages). 

#### 2.2.3. Ce-SANP Nanoparticle Formulation

Ce-SANP were prepared by simple mixing the components (inorganic nanoparticles and liposomes) at room temperature in a 1:1 *v/v* ratio followed by incubation for 25 min. 

#### 2.2.4. Nanoparticle Physico-Chemical Characterization

The formulations were characterized in terms of colloidal dimensions, polydispersity index (PDI), surface charge, and stability against aggregation in water by using dynamic light scattering (DLS) (Zetasizer Nano Z, Malvern, UK). Prior to the measurements, samples were diluted to 1% in filtered deionized water. For each formulation, the z-average diameter, PDI, and zeta potential were calculated as mean ± standard deviation of measurements from N ≥ 2 independent batches. 

#### 2.2.5. Scanning Electron Microscopy and Energy Dispersive X-ray Spectroscopy (SEM-EDX)

Microstructural and chemical analyses of the nanomaterials were were performed at the scanning electron microscopy lab of the Dept. of Earth Sciences, Environment and Resources, Federico II University, Napoli—Italy. A Field Emission Scanning Electron Microscope equipped with an Energy Dispersive Spectrometer (FESEM/EDS; Zeiss Merlin VP Compact coupled with Oxford Instruments Microanalysis Unit; Carl-Zeiss-Strasse, Oberkochen, Germany) was used for observations and spot analyses. Data sets were obtained using an INCA X-stream pulse processor (Oberkochen, Germany) (15-kV primary beam voltage, 50–100 A filament current, variable spot size, from 30,000 to 200,000× magnification, 20 mm working distance, and 50 s real-time counting) by means of INCA Energy software 5.05 (XPP array and pulse pile-up corrections). Optimization of signals was carried out using cobalt (FWHM peak height of the strobed zero = 60–65 eV) as a reference. Smithsonian Institute and MAC (Micro-Analysis Consultants Ltd., Saint Ives, UK) standard materials were used for element calibration: Cerium Phosphate (Ce), diopside (Ca), apatite (P). Accuracies about EDS chemical analyses are reported in [22].

#### 2.2.6. Peroxidase Assay

To assess the peroxidase-like activity of Ce-CaP NPs and Ce-SANP, 50 μL of the TMB substrate solution was added to each well of a clear 96-well plate and mixed with 50 μL of an aqueous suspension of Ce-CaP NPs or Ce-SANP at various concentrations. The reaction mixture was incubated for 1 min and was stopped by the addition of 50 μL of stop solution (0.16 M sulfuric acid). The absorbance was measured on a spectrophotometer (Thermo Scientific™ Multiskan™ GO Microplate Spectrophotometer) at 451 nm. 

#### 2.2.7. Superoxide Dismutase (SOD) Assay

The SOD-like activity of Ce-CaP NPs and Ce-SANP was assessed with an SOD assay kit (cat. n. 19,160) following the manufacturer’s instructions. Briefly, 10 μL of an aqueous suspension of Ce-CaP NPs or Ce-SANP at various concentrations was added to 100 μL of an aqueous solution containing the substrate, 2-(4-iodophenyl)-3-(4-nitrophenyl)-5-(2,4-disulfophenyl)-2H-tetrazolium, monosodium salt (WST-1) to each well of a clear 96-well plate. A total of 10 μL of a xanthine oxidase solution (obtained by diluting 15 μL of xanthine oxidase solution in 2.5 mL of dilution buffer) was then added to each well and the plate was incubated at 37 °C for 20 min. The absorbance was then measured at 440 nm by using a GloMax^®^ Discover Microplate Reader (Promega Instruments). As a control, 10 μL of DI water was added to wells containing the WST-1 solution and the enzyme solution (Blank 1, full conversion of the WST-1 substrate) or to wells containing the WST-1 solution and the dilution buffer (Blank 2, no conversion). The SOD activity was calculated by using the following formula:

$$SOD\;activity\;\%=\frac{(A_{Blank1}-A_{Blank2})-A_{Sample}}{A_{Blank1}-A_{Blank2}}\times 100,$$

where $A_{Blank1}$, $A_{Blank2}$, and $A_{Sample}$ are the measured absorbance values of wells containing the Blank 1, the Blank 2 or the Ce-CaP NPs/Ce-SANP, respectively.

#### 2.2.8. Biological Characterization of Ce-SANP on Mitochondrial Function

Use of Experimental Animals

All the experiments were performed in accordance with the guidelines of the Declaration of Helsinki and with the procedures described in experimental protocols approved by the Ethical Committee of “Federico II” University of Naples, Naples, Italy (protocol 119, May 2022), and by the Italian Ministry of Health (D. Lgs. March 4th 2014 from Italian Ministry of Health and DIR 2010/63 from UE). 

#### 2.2.9. Cell Culture

Primary Cultures of Rat Cortical Neurons

Cortical neurons were obtained from the brains of 14/16-day-old Wistar rat embryos, dissected and cultured as previously reported [23]. Cytosine-β-D-Arabino-furanoside (Ara-C) (10 μM) was added at 3 days in vitro (DIV) to prevent the growth of non-neuronal cells. For the experiments, cortical neurons were used at 7–10 DIV. In consideration of the experimental procedures, the characterization of these neuronal cultures showed that at day 7, cell purity was ~98%.

Clonal Cells

Rat pheochromocytoma (PC12) cells were cultured and differentiated with nerve growth factor (NGF, 50 ng/mL) as previously described [24]. 

#### 2.2.10. Chemical Hypoxia and Reoxygenation

Chemical hypoxia was obtained by adding to the PC12 cells 5 μg/mL oligomycin (an oxidative phosphorylation, OXYPHOS, inhibitor) plus 2 mM 2-deoxy-D-glucose (a glycolysis inhibitor) in a glucose-free medium for 45 min. Reoxygenation was performed by incubating cells in a culture medium containing normal levels of glucose for 3 h [25]. These conditions reproduced a typical hypoxia occurring during brain ischemia. 

#### 2.2.11. Oxygen and Glucose Deprivation Followed by Reoxygenation (OGD/Rx)

Ischemic insult was reproduced in vitro by exposing neurons to 3 h of oxygen and glucose deprivation (OGD) performed in a glucose-free medium previously saturated with 95% N_2_ and 5% CO_2_. Hypoxic conditions were maintained using a hypoxia chamber (temperature 37 °C, atmosphere 5% CO_2_ and 95% N_2_). At the end of incubation, the glucose-free medium was replaced with a culture medium containing normal levels of O_2_ and glucose; thus, reoxygenation was achieved by returning neurons to normoxic conditions (5% CO_2_ and 95% air, temperature 37 °C) for 24 h [23].

#### 2.2.12. Analysis of Mitochondrial activity

Mitochondrial activity was assessed by the 3[4,5-dimethylthiazol-2-yl]-2,5-diphenyl-tetrazolium bromide (MTT) assay. After treatments, primary cortical neurons and neuronal PC12 cells were incubated with MTT for 1 h at 37 °C. Then, samples were collected in dimethyl sulfoxide (DMSO) and measured spectrophotometrically at 540 nm. Data were expressed as a percentage of cell viability compared to control cultures.

#### 2.2.13. Measurement of ROS on Single-Cell

Neurons were seeded on glass coverslips and exposed to hypoxic conditions in the absence or presence of Ce-SANP. Then, neurons were incubated with 2′,7′-dichlorodihydrofluorescein diacetate (DCFH-DA, 17.5 µM) as previously described [26]. Using the same imaging system described in [Ca^2+^]_i_ measurement, each coverslip was exposed at 485 nm excitation for 10 s and the emitted light was passed through a 530 nm barrier filter. ROS level was expressed as DCF fluorescence in arbitrary units.

#### 2.2.14. Quantification of ATP Level

ATP content was measured by a commercial bioluminescent assay (ATP bioluminescent assay kit, Merck) as previously described [27]. After treatments, ATP was extracted by boiling samples for 1 min in a solution containing 100 mM TRIS, 4 mM EDTA, pH 7.75. Bioluminescence measurements were carried out on 100 μL of each sample mixed with 100 μL of luciferin-luciferase solution using a standard luminometer. ATP content was calculated using a standard curve obtained by serial dilution of 2 μM ATP standard solution.

#### 2.2.15. [Ca^2+^]_i_ Measurement 

[Ca^2+^]_i_ was measured by single-cell computer-assisted video-imaging. Briefly, primary cortical neurons and PC12 cells, seeded on glass coverslips, were loaded with 10 µM Fura-2 acetoxymethyl ester (Fura-2AM) for 30 min at 37 °C in normal Krebs solution containing the following (in mM): 5.5 KCl, 160 NaCl, 1.2 MgCl_2_, 1.5 CaCl_2_, 10 glucose, and 10 Hepes–NaOH, pH 7.4. Then, coverslips were placed into a perfusion chamber mounted onto a Zeiss Axiovert 200 microscope (Carl Zeiss, Germany) connected to MicroMax 512BFT cooled CCD camera (Princeton Instruments, Trenton, NJ, USA) and a LAMBDA 10-2 filter wheeler (Sutter Instruments, Novato, CA, USA). After excitation at 340 and 380 nm, the fluorescence intensity of Fura-2 was collected and [Ca^2+^]_i_ was measured every 3 s by Grynkiewicz’s equation [28], whose parameters were determined for individual cells, as previously described [29]. [Ca^2+^]_i_ was expressed in the nanomolar range by using Meta-Morph/MetaFluor Imaging System software (Universal Imaging, West Chester, PA) and applying the equation: [Ca^2+^]_i_ = KD F0 (R − Rmin)/FS (Rmax − R), where KD is the apparent equilibrium dissociation constant for Fura-2 at 225 nM. The amount of Ca^2+^ extruded in the cytoplasm upon FCCP (1µM) exposure is widely considered as the index of the mitochondrial Ca^2+^ efflux. 

#### 2.2.16. Statistical Analysis

Data are expressed as mean ± S.E.M. The statistical analysis was performed with an unpaired one-way analysis of variance followed by the Newman–Keuls test. Statistical significance was accepted at the 95% confidence level (*p* < 0.05).

## 3. Results and Discussion

### 3.1. Synthesis of Cerium-CaP Nanoparticles (Ce-CaP NPs)

**Figure 1:**
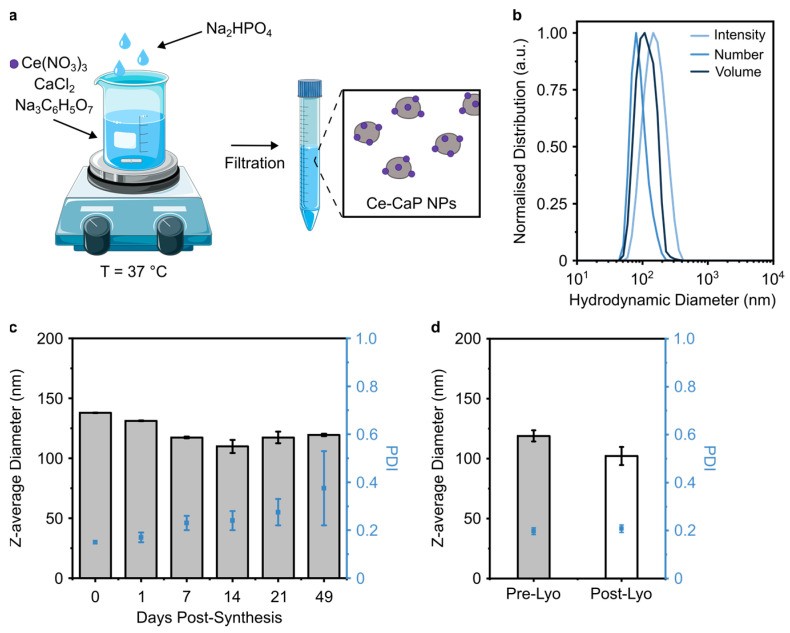
Synthesis and the physico-chemical characterization of Ce-CaP NPs. (**a**) Schematic of the synthesis of Ce-CaP NPs; the schematic was partly generated using Servier Medical Art, provided by Servier, licensed under a Creative Commons Attribution 3.0 unported license. (**b**) Normalized intensity (light blue), number (blue), and volume (dark blue) distributions of the Ce-CaP NPs hydrodynamic diameter as measured by DLS. Z-average diameter and PDI of Ce-CaP NPs at 4 °C over 49 days (**c**) and pre- and post-lyophilization (**d**). Data are shown as mean ± standard deviation of N ≥ 2 independent batches.

Calcium phosphate nanoparticles (CaP NPs) have been widely investigated for drug delivery applications due to their high biocompatibility, pH-dependent dissolution behavior and ability to complex various payloads, including proteins and nucleic acids [30]. CaP NPs can also be doped with other metal ions such as iron, gadolinium, or europium for, e.g., magnetic resonance imaging applications [30]. In order to obtain lipid SANP with ROS-scavenging properties, we first modified their CaP core with cerium ions by adapting a well-established protocol for CaP nanoparticles synthesis. This protocol entails the mixing of two aqueous solutions containing the calcium and phosphate precursors at basic pH [21]. In this work, hybrid cerium-CaP nanoparticles (Ce-CaP NPs) were prepared by mixing an aqueous solution containing Ce (NO_3_)_3_, CaCl_2_ and trisodium citrate with an aqueous solution containing Na_2_HPO_4_ followed by incubation at 37 °C and filtration (Figure 1a). The sodium citrate was added as a stabilizer due to its ability to control the crystallization of both CaP [31] and cerium oxide [32] nanoparticles, while an excess of Ce^3+^ with respect to PO_4_^3−^ was used to ensure the formation of cerium oxide alongside cerium phosphate [12].

The obtained Ce-CaP NPs were characterized in terms of their colloidal dimensions via dynamic light scattering (DLS) and exhibited a z-average diameter of 136.50 ± 2.26 nm and a polydispersity index (PDI) of 0.16 ± 0.02 (Figure 1b). We also carried out DLS measurements to characterize their colloidal stability against aggregation over 49 days at 4 °C. The Ce-CaP NPs had good colloidal stability in water over 21 days, with negligible differences in the z-average diameter, while a gradual increase in the PDI was detected (Figure 1c). We, therefore, investigated the possibility to lyophilize these formulations and we observed minimal variations in the z-average diameter and PDI following the reconstitution of the lyophilized formulations (Figure 1d), thus suggesting that lyophilization may be a viable strategy to extend their shelf life.

### 3.2. SEM-EDS Analysis of Ce-CaP Nanoparticles

**Figure 2:**
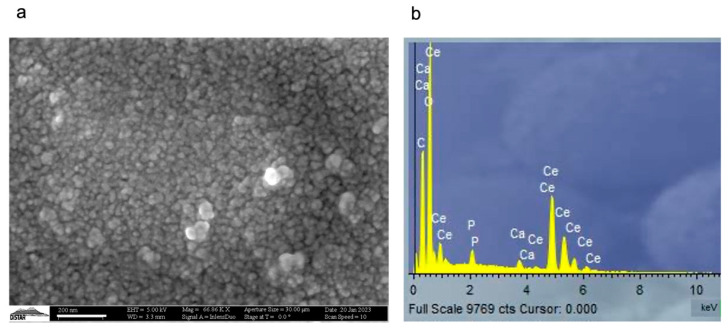
Structural and compositional characterization of Ce-CaP NPs. Representative SEM micrograph (**a**) and EDS spectrum (**b**) of Ce-CaP NPs; scale bar: 200 nm.

We next performed SEM-EDX analysis to gain further insights into the structural and compositional features of the Ce-CaP NPs, which exhibited a spherical shape and sizes ranging between 25 and 130 nm (Figure 2a). The discrepancies in the nanoparticle size measured by SEM and DLS may be ascribed to aggregation phenomena occurring in water, in analogy to what has been observed for titanium oxide nanoparticles [33]. EDS analysis revealed that Ce-CaP NPs comprise cerium at 92.5 ± 0.4%, calcium at 2.3 ± 0.1%, and phosphorus at 5.2 ± 0.4% (Figure 2b), which may suggest the coexistence of cerium phosphate and cerium oxide. It has been previously hypothesized that upon the dissolution of the precursor salts, Ce^3+^ and PO_4_^3−^ form cerium phosphate while the excess Ce^3+^ ions reacts with dissolved oxygen to form cerium oxide [12].

### 3.3. Characterization of Ce-CaP Nanoparticles: Enzyme-Like Activity

**Figure 3:**
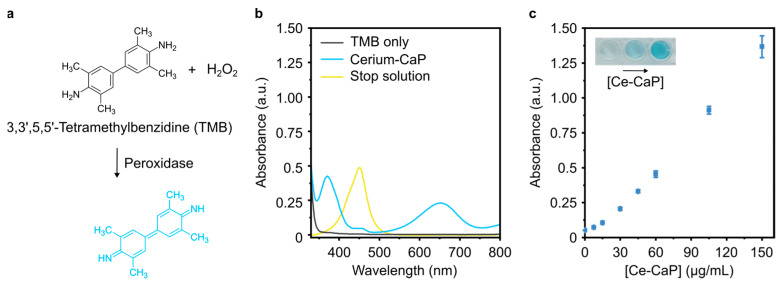
Peroxidase-like activity of Ce-CaP NPs. (**a**) Schematic of the redox reaction between TMB, H2O2, and peroxidase. (**b**) Absorption spectra of samples containing the TMB substrate solution only (grey trace), TMB substrate solution and Ce-CaP NPs at a concentration of 56 μg/mL (blue trace), or TMB substrate solution, Ce-CaP NPs at a concentration of 56 μg/mL, and 0.16 M sulfuric acid (stop solution, yellow trace). (**c**) Absorbance of the reaction product measured at 451 nm with varying concentrations of the Ce-CaP NPs. Ce-CaP NPs were added to the TMB substrate solution and the reaction was stopped after 1 min with the addition of a stop solution. Data points are represented as mean ± s.d. (N = 3 independent batches, n = 3 technical replicates). The inset shows the color change due to TMB oxidation with increasing concentration of Ce-CaP NPs.

Previous studies have demonstrated the ability of cerium oxide nanoparticles to mimic the activity of ROS-scavenging enzymes such as peroxidase and SOD, potentially due to a continuous redox cycle of Ce^3+^ and Ce^4+^ and the presence of oxygen vacancies at the surface of the nanoparticles [34]. In order to investigate whether the Ce-CaP NPs exhibited peroxidase-like activity, we used a colorimetric assay based on the redox reaction between 3,3′,5,5′-Tetramethylbenzidine (TMB) and H_2_O_2_ (Figure 3a) [11]. In the presence of Ce-CaP NPs and H_2_O_2_, a blue-colored, cationic radical derivative of TMB with characteristic absorption peaks at 370 and 653 nm (Figure 3b, blue trace) was obtained; no absorption peaks could be detected when only TMB and H_2_O_2_ were present (Figure 3b, grey trace). The reaction could be stopped by the addition of a solution containing 0.16 M sulfuric acid (“stop solution”), which converted the cationic radical TMB derivative into a yellow diamine compound with a characteristic absorption peak at 451 nm (Figure 3b, yellow trace) [35]. The addition of Ce-CaP NPs at various concentrations to a TMB substrate solution resulted in an increase in the absorbance at 451 nm, which corresponded to higher concentrations of the diamine derivative of TMB (Figure 3c). These findings indicated that the Ce-CaP NPs could work as peroxidase mimics.

**Figure 4:**
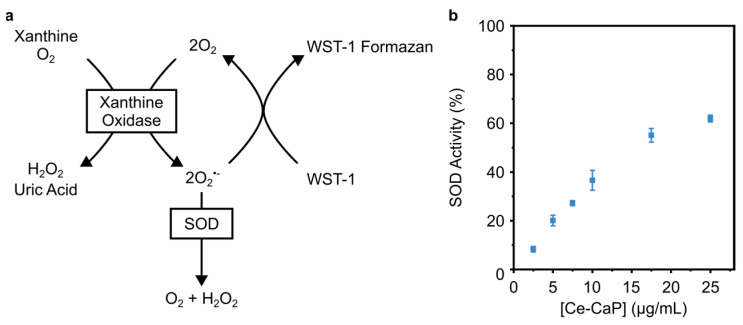
SOD-like activity of Ce-CaP NPs. (**a**) Schematic of the assay used to quantify the SOD-like activity of Ce-CaP NPs. (**b**) SOD-like activity of Ce-CaP NPs as a function of their concentration. Ce-CaP NPs were added to a solution containing xanthine oxidase and tetrazolium salt; the mixture was incubated at 37 °C for 20 min, after which the absorbance at 450 nm was recorded. Data points are represented as mean ± s.d. (N = 3 independent batches, n = 3 technical replicates).

We next probed the SOD-like activity of Ce-CaP NPs with a colorimetric assay based on the oxidation of a water-soluble tetrazolium salt, which is converted into a formazan dye with an absorption peak at 450 nm by superoxide anions generated by xanthine oxidase. In the presence of SOD, the superoxide anion is converted into hydrogen peroxide and oxygen, leading to a decrease in the concentration of the dye (Figure 4a). The SOD activity can be regarded as inhibition activity since higher concentrations of SOD lead to a more pronounced decrease in the measured absorbance [36]. The Ce-CaP NPs exhibited SOD-like activity and the addition of increasing concentrations of Ce-CaP NPs led to an increase in the SOD-like activity (Figure 4b), similar to what has been previously reported for cerium oxide nanoparticles [36,37,38].

### 3.4. Formulation of Ce-SANP

**Figure 5:**
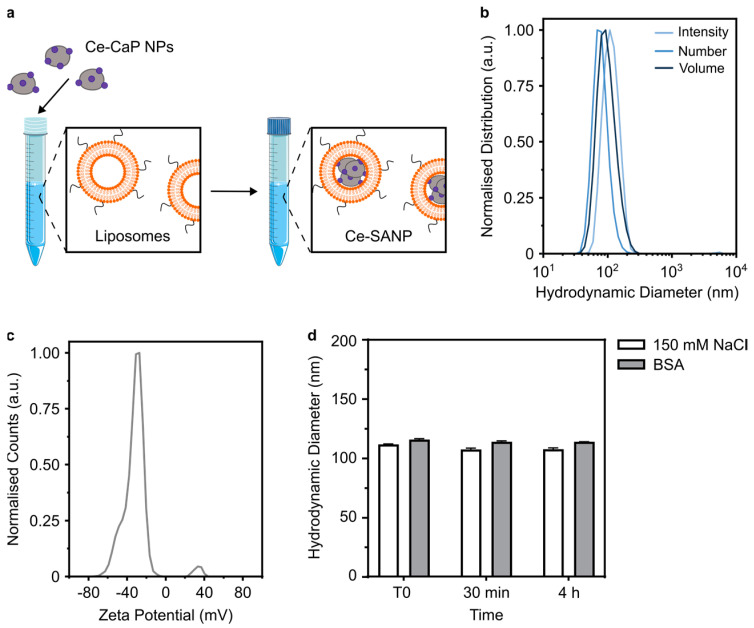
Physico-chemical characterization of Ce-SANP. (**a**) Schematic of the synthesis of Ce-SANP; the schematic was partly generated using Servier Medical Art, provided by Servier, licensed under a Creative Commons Attribution 3.0 unported license. (**b**) Normalized intensity (light blue), number (blue), and volume (dark blue) distributions of the Ce-SANP hydrodynamic diameter as measured by DLS. (**c**) Zeta potential measurement and (**d**) stability against aggregation at 37 °C of the Ce-SANP formulations following dilution in 150 mM NaCl or BSA. Data are represented as mean ± s.d. (N = 3 independent batches, n = 3 technical replicates).

Coating metal oxide nanoparticles with biomaterials has been proposed as a strategy to improve nanoparticle serum stability against aggregation and cellular uptake, as well as to provide a handle for surface functionalization with, e.g., targeting moieties [39]. Cerium-based nanoparticles have been coated with various materials such as dextran/poly (acrylic) acid [40,41], heparin [42], PEG-based copolymers [38], or cell-membrane derived lipids [43]. In this work, we assessed the possibility to form SANP by mixing Ce-CaP NPs with DOTAP:CerPEG liposomes to obtain lipid-coated Ce-CaP NPs (Figure 5a). The liposomes exhibited a z-average diameter of 135 ± 3.34 nm, a PDI of 0.037 ± 0.02, and a zeta potential of + 45.09 ± 1.72 mV. Mixing the liposomes with the Ce-CaP NPs yielded SANP with a z-average diameter of 108.93 ± 1.62 nm and a PDI of 0.11 ± 0.06 (Figure 5b); the Ce-SANP were shown to be negatively charged, with a zeta potential of −30.98 ± 1.34 mV (Figure 5c). A reduction in the z-average diameter was observed for the Ce-SANP compared to the liposome and the Ce-CaP NPs, which may suggest a structural reorganization of these components following mixing to obtain Ce-SANP, as observed in previous studies [44].

We also tested the stability against aggregation of the Ce-SANP formulations upon a 4 h incubation in a 1 *w/v* % BSA solution at 37 °C to evaluate their suitability for intravenous administration. The mean hydrodynamic diameter of the Ce-SANP formulations was measured via DLS at T0, 30 min and 4 h post-incubation in a BSA solution; the nanoparticles were also incubated in a 150 mM NaCl solution as a positive control. A negligible increase in the hydrodynamic diameter was observed following incubation with BSA compared to the control and this value remained unchanged over time, suggesting that the Ce-SANP formulations have good stability against aggregation at 37 °C in the presence of serum proteins (Figure 5d). This is probably due to the presence of the PEGylated lipid in the nanoparticle composition which effectively prevents nanoparticle aggregation and protein adsorption on the surface [45].

### 3.5. Enzyme-Like Activity of Ce-SANP

**Figure 6:**
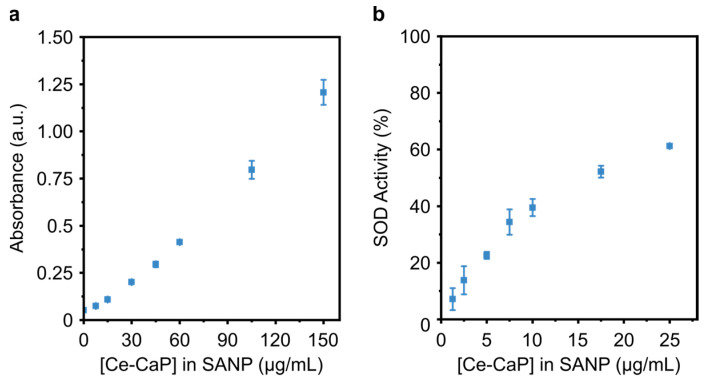
Peroxidase- and SOD-like activity of Ce-SANP. (**a**) Absorbance of the reaction product measured at 451 nm with varying concentrations of Ce-SANP. Ce-SANP were added to the TMB substrate solution and the reaction was stopped after 1 min with the addition of a stop solution. Data points are represented as mean ± s.d. (N = 5 independent batches, n = 3 technical replicates). (**b**) SOD-like activity of Ce-SANP as a function of their concentration. Ce-SANP were added to a solution containing xanthine oxidase and tetrazolium salt; the mixture was incubated at 37 °C for 20 min, after which the absorbance at 450 nm was recorded. Data points are represented as mean ± s.d. (N = 3 independent batches, n = 3 technical replicates).

In order to understand the effect of the lipid coating on the enzyme-like properties of Ce-CaP NPs, we carried out the peroxidase and the SOD activity assays on the Ce-SANP by using an equivalent concentration of Ce-CaP NPs. The Ce-SANP retained their peroxidase-like activity (Figure 6a), which was a function of the concentration of Ce-CaP NPs in the SANP formulation. Ce-SANP also exhibited similar SOD-like activity to bare Ce-CaP NPs with a concentration-dependent behavior. Overall, the lipid coating did not impair the enzyme-like properties of the Ce-CaP NPs, as previously observed for cerium oxide nanoparticles encapsulated in nanostructured lipid carriers [46] or adsorbed on the surface of mesoporous silica nanoparticles which were subsequently coated with a lipid shell [47].

### 3.6. Effect of Ce-SANP on Mitochondrial Activity and Function in Primary Cortical Neurons Exposed to OGD Followed by Reoxygenation and Differentiated PC12 Cells Exposed to Chemical Hypoxia

**Figure 7:**
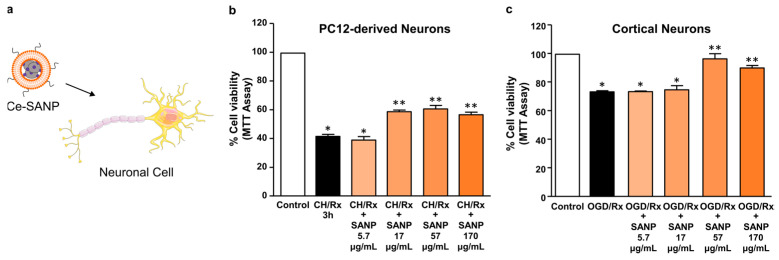
Effects of Ce-SANP on mitochondrial activity in primary cortical neurons exposed to OGD followed by reoxygenation and differentiated PC12 cells exposed to chemical hypoxia. Ce-SANPs were added to neuronal cells under oxidative stress conditions (**a**) at various concentrations (the schematic was partly generated using Servier Medical Art, provided by Servier, licensed under a Creative Commons Attribution 3.0 unported license). Dose-dependent effects of Ce-SANP on cell viability, measured as mitochondrial activity, in (**b**) PC12 cells differentiated with NGF and exposed to chemical hypoxia followed by reoxygenation (CH/Rx) and (**c**) primary cortical neurons exposed to OGD followed by reoxygenation (OGD/Rx). In both cases, Ce-SANPs were added only during the reoxygenation phase. The white bars represent cells treated with vehicle while the black bars represent the cells under oxidative stress conditions without the addition of Ce-SANP. * *p* < 0.05 vs. control, ** *p* < 0.05 vs. CH/Rx or OGD/Rx and vs. CH/Rx + SANP at the lowest concentration (5.7 µg/mL or OGD/Rx + SANP at the lowest concentration (5.7 µg/mL).

Alterations in mitochondrial function are increasingly recognized in neurological diseases. Given the favorable colloidal properties and enzyme-mimetic activity of the Ce-SANP formulations, we assessed their ability to rescue neurons from mitochondrial damage induced by the exposure to ischemia-like stimuli. Ce-SANP were added to the cells during the reoxygenation phase at concentrations between 5.7 and 170 μg/mL and were able to improve cellular viability, in a dose-dependent manner, of primary cortical neurons exposed to OGD followed by reoxygenation and of differentiated PC12 cells exposed to chemical hypoxia plus reoxygenation (Figure 7). This latter model was obtained by treating neuronal cells with OXYPHOS and glycolysis inhibitors which mimic the hypoxic conditions of brain ischemia.

**Figure 8:**
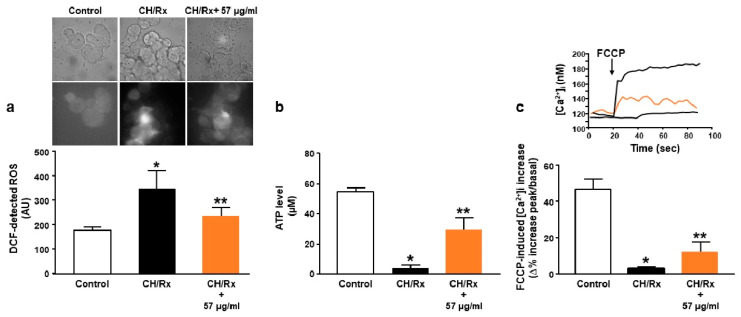
Effects of Ce-SANP on mitochondrial function (e.g., ROS production, ATP levels, mitochondrial Ca^2+^ homeostasis). (**a**) Arbitrary units of fluorescence in NGF-differentiated PC12 cells incubated with DCFH-DA under control conditions, after chemical hypoxia + reoxygenation alone and chemical hypoxia + reoxygenation in the presence of Ce-SANP (57 µg/mL). Ce-SANP were added only during the reoxygenation phase. On the top of the panel, representative images for each treatment were reported. The bars show mean values ± SEM from three different experimental sessions. * *p* ≤ 0.05 vs. control; ** *p* ≤ 0.01 vs. CH/Rx. (**b**) basal ATP content (in µM) in differentiated PC12 cells under control conditions, after chemical hypoxia + reoxygenation alone and chemical hypoxia + reoxygenation in the presence of Ce-SANP (57 µg/mL). Ce-SANP were added only during the reoxygenation phase. The bars show mean values ± SEM from three different experimental sessions. * *p* ≤ 0.05 vs. control; ** *p* ≤ 0.01 vs. CH/Rx. (**c**) FCCP-induced [Ca^2+^]i increase in NGF-differentiated PC12 cells under control conditions, after chemical hypoxia + reoxygenation alone and chemical hypoxia + reoxygenation in the presence of Ce-SANP (57 µg/mL). Ce-SANP were added only during the reoxygenation phase. On the top of the panel, representative traces for the effects of each treatment on [Ca^2+^]_i_ are reported.

We further measured several mitochondrial parameters including ATP production, calcium transport, and ROS generation in the presence of the Ce-SANP formulation. Ce-SANP at a concentration of 57 μg/mL were able to significantly prevent: (a) hypoxia-induced ROS production; (b) decrease in ATP level; (c) mitochondrial Ca^2+^ homeostasis dysfunction, which was detected as a rapid reduction in FCCP-induced Ca^2+^ release (Figure 8). In particular, the present data clearly showed a protective mechanism of these nanoparticles, aiming to preserve the mitochondrial function, which was highly compromised in neurons exposed to hypoxic conditions during the ischemic insult. The fact that Ce-SANP preserved ATP content is of exceptional importance and is testified by the partial reestablishment of mitochondrial Ca^2+^ transport. In addition, it should be also taken into account that many plasma membrane and organellar pumps moving Ca^2+^ are ATP-dependent. In fact, a lack of ATP, occurring under hypoxic conditions, triggers intracellular Ca^2+^ concentration dysfunction, leading to the activation of several cell death pathways. With regard to the internalization of cerium-doped SANP in neurons, it has been shown that endocytosis is the privileged mechanism allowing for the targeted delivery of drugs. For instance, nanoparticles based on selenium enter cells by activating clathrin-associated endocytosis [48], the major vesicle retrieval mechanism in neurons [49]. These results highlighted the potential of Ce-SANP as a well-suited formulation of Ce, useful for the treatment of the oxidative stress associated with neurodegenerative diseases.

## 4. Conclusions

In this work, we developed cerium-doped SANP for the treatment of ischemia-like conditions reproduced in vitro by exposing cortical neurons to OGD followed by reoxygenation or differentiated PC12 cells to specific blockers of mitochondrial oxidative phosphorylation and glycolysis. We engineered the SANP inorganic core with cerium by synthesizing cerium-doped CaP NPs, which had hydrodynamic diameters < 150 nm and low PDI, as well as peroxidase- and SOD-like activity. Upon mixing with cationic liposomes, we obtained cerium-doped SANP, which retained the enzyme-mimicking activity of the Ce-CaP NPs and were able to protect PC12 differentiated neurons and primary cortical neurons from ischemia-induced cell death in a dose-dependent manner. Furthermore, they effectively prevented hypoxia-induced ROS production, a decrease in ATP levels, and mitochondrial Ca^2+^ homeostasis dysfunction. These results highlighted the ability of these antioxidant delivery systems to preserve mitochondrial functions as ATP production and Ca^2+^ storing. Future work will entail loading the cerium-doped SANP with drugs commonly used for the treatment of neurodegenerative diseases and evaluating the putative synergic therapeutic efficacy in vitro and *in vivo*. This work paves the way for the development of an entirely new class of “bioactive” nanoparticles with the potential to enable multi-modal therapy for the treatment of neurodegenerative diseases.

## Data Availability

The data presented in this study are available on request from the corresponding author.

## References

[1] Gan L., Cookson M.R., Petrucelli L., la Spada A.R. (2018). Converging Pathways in Neurodegeneration, from Genetics to Mechanisms. Nat. Neurosci..

[2] Powers W.J. (2020). Acute Ischemic Stroke. N. Engl. J. Med..

[3] Hou Y., Dan X., Babbar M., Wei Y., Hasselbalch S.G., Croteau D.L., Bohr V.A. (2019). Ageing as a Risk Factor for Neurodegenerative Disease. Nat. Rev. Neurol..

[4] Forman M.S., Trojanowski J.Q., Lee V.M.-Y. (2004). Neurodegenerative Diseases: A Decade of Discoveries Paves the Way for Therapeutic Breakthroughs. Nat. Med..

[5] Andersen J.K. (2004). Oxidative Stress in Neurodegeneration: Cause or Consequence?. Nat. Med..

[6] Martinelli C., Pucci C., Battaglini M., Marino A., Ciofani G. (2020). Antioxidants and Nanotechnology: Promises and Limits of Potentially Disruptive Approaches in the Treatment of Central Nervous System Diseases. Adv. Health Mater..

[7] Wojsiat J., Zoltowska K.M., Laskowska-Kaszub K., Wojda U. (2018). Oxidant/Antioxidant Imbalance in Alzheimer’s Disease: Therapeutic and Diagnostic Prospects. Oxid. Med. Cell. Longev..

[8] Yang B., Chen Y., Shi J. (2019). Nanocatalytic Medicine. Adv. Mater..

[9] Naz S., Beach J., Heckert B., Tummala T., Pashchenko O., Banerjee T., Santra S. (2017). Cerium Oxide Nanoparticles: A ‘Radical’ Approach to Neurodegenerative Disease Treatment. Nanomedicine.

[10] Xiao G., Li H., Zhao Y., Wei H., Li J., Su H. (2022). Nanoceria-Based Artificial Nanozymes: Review of Materials and Applications. ACS Appl. Nano. Mater..

[11] Wang W., Jiang X., Chen K. (2012). CePO4:Tb,Gd Hollow Nanospheres as Peroxidase Mimic and Magnetic–Fluorescent Imaging Agent. Chem. Commun..

[12] Vinothkumar G., Lalitha A.I., Suresh Babu K. (2019). Cerium Phosphate–Cerium Oxide Heterogeneous Composite Nanozymes with Enhanced Peroxidase-Like Biomimetic Activity for Glucose and Hydrogen Peroxide Sensing. Inorg. Chem..

[13] Shcherbakov A.B., Zholobak N.M., Baranchikov A.E., Ryabova A.V., Ivanov V.K. (2015). Cerium Fluoride Nanoparticles Protect Cells against Oxidative Stress. Mater. Sci. Eng. C.

[14] Choi S.W., Kim J. (2020). Recent Progress in Autocatalytic Ceria Nanoparticles-Based Translational Research on Brain Diseases. ACS Appl. Nano. Mater..

[15] Sun J., Roy S. (2021). Gene-Based Therapies for Neurodegenerative Diseases. Nat. Neurosci..

[16] Martier R., Konstantinova P. (2020). Gene Therapy for Neurodegenerative Diseases: Slowing Down the Ticking Clock. Front. Neurosci..

[17] Leavitt B.R., Tabrizi S.J. (2020). Antisense Oligonucleotides for Neurodegeneration. Science.

[18] Roberts T.C., Langer R., Wood M.J.A. (2020). Advances in Oligonucleotide Drug Delivery. Nat. Rev. Drug Discov..

[19] Porru M., Zappavigna S., Salzano G., Luce A., Stoppacciaro A., Balestrieri M.L., Artuso S., Lusa S., de Rosa G., Leonetti C. (2014). Medical Treatment of Orthotopic Glioblastoma with Transferrin-Conjugated Nanoparticles Encapsulating Zoledronic Acid. Oncotarget.

[20] Salzano G., Zappavigna S., Luce A., D’Onofrio N., Balestrieri M.L., Grimaldi A., Lusa S., Ingrosso D., Artuso S., Porru M. (2016). Transferrin-Targeted Nanoparticles Containing Zoledronic Acid as a Potential Tool to Inhibit Glioblastoma Growth. J. Biomed Nanotechnol..

[21] Campani V., Zappavigna S., Scotti L., Abate M., Porru M., Leonetti C., Caraglia M., de Rosa G. (2020). Hybrid Lipid Self-Assembling Nanoparticles for Brain Delivery of MicroRNA. Int. J. Pharm..

[22] Rispoli C., De Bonis A., Guarino V., Graziano S.F., Di Benedetto C., Esposito R., Morra V., Cappelletti P. (2019). The ancient pozzolanic mortars of the Thermal complex of Baia (Campi Flegrei, Italy). J. Cult. Herit..

[23] Secondo A., Petrozziello T., Tedeschi V., Boscia F., Vinciguerra A., Ciccone R., Pannaccione A., Molinaro P., Pignataro G., Annunziato L. (2019). ORAI1/STIM1 Interaction Intervenes in Stroke and in Neuroprotection Induced by Ischemic Preconditioning Through Store-Operated Calcium Entry. Stroke.

[24] Pannaccione A., Secondo A., Scorziello A., Calì G., Taglialatela M., Annunziato L. (2005). Nuclear Factor-ΚB Activation by Reactive Oxygen Species Mediates Voltage-Gated K+ Current Enhancement by Neurotoxic β-Amyloid Peptides in Nerve Growth Factor-Differentiated PC-12 Cells and Hippocampal Neurones. J. Neurochem..

[25] Secondo A., Staiano R.I., Scorziello A., Sirabella R., Boscia F., Adornetto A., Valsecchi V., Molinaro P., Canzoniero L.M.T., di Renzo G. (2007). BHK Cells Transfected with NCX3 Are More Resistant to Hypoxia Followed by Reoxygenation than Those Transfected with NCX1 and NCX2: Possible Relationship with Mitochondrial Membrane Potential. Cell Calcium..

[26] Petrozziello T., Secondo A., Tedeschi V., Esposito A., Sisalli M., Scorziello A., di Renzo G., Annunziato L. (2017). ApoSOD1 Lacking Dismutase Activity Neuroprotects Motor Neurons Exposed to Beta-Methylamino-L-Alanine through the Ca2+/Akt/ERK1/2 Prosurvival Pathway. Cell Death Differ..

[27] Cocco S., Secondo A., del Viscovo A., Procaccini C., Formisano L., Franco C., Esposito A., Scorziello A., Matarese G., di Renzo G. (2015). Polychlorinated Biphenyls Induce Mitochondrial Dysfunction in SH-SY5Y Neuroblastoma Cells. PLoS ONE.

[28] Grynkiewicz G., Poenie M., Tsien R.Y. (1985). A New Generation of Ca2+ Indicators with Greatly Improved Fluorescence Properties. J. Biol. Chem..

[29] Urbanczyk J., Chernysh O., Condrescu M., Reeves J.P. (2006). Sodium-Calcium Exchange Does Not Require Allosteric Calcium Activation at High Cytosolic Sodium Concentrations. J. Physiol..

[30] Cai A.-Y., Zhu Y.-J., Qi C. (2020). Biodegradable Inorganic Nanostructured Biomaterials for Drug Delivery. Adv. Mater. Interfaces.

[31] di Mauro V., Iafisco M., Salvarani N., Vacchiano M., Carullo P., Ramírez-Rodríguez G.B., Patrício T., Tampieri A., Miragoli M., Catalucci D. (2016). Bioinspired Negatively Charged Calcium Phosphate Nanocarriers for Cardiac Delivery of MicroRNAs. Nanomedicine.

[32] Heckman K.L., DeCoteau W., Estevez A., Reed K.J., Costanzo W., Sanford D., Leiter J.C., Clauss J., Knapp K., Gomez C. (2013). Custom Cerium Oxide Nanoparticles Protect against a Free Radical Mediated Autoimmune Degenerative Disease in the Brain. ACS Nano.

[33] Kignelman G., Eyley S., Zhou C., Tunca B., Gonon M., Lahem D., Seo J.W., Thielemans W. (2021). Colloidal Stability and Aggregation Mechanism in Aqueous Suspensions of TiO _2_ Nanoparticles Prepared by Sol–Gel Synthesis. Langmuir.

[34] Yang Y., Mao Z., Huang W., Liu L., Li J., Li J., Wu Q. (2016). Redox Enzyme-Mimicking Activities of CeO2 Nanostructures: Intrinsic Influence of Exposed Facets. Sci. Rep..

[35] Alizadeh N., Salimi A., Sham T.-K., Bazylewski P., Fanchini G. (2020). Intrinsic Enzyme-like Activities of Cerium Oxide Nanocomposite and Its Application for Extracellular H _2_ O _2_ Detection Using an Electrochemical Microfluidic Device. ACS Omega.

[36] Baldim V., Bedioui F., Mignet N., Margaill I., Berret J.-F. (2018). The Enzyme-like Catalytic Activity of Cerium Oxide Nanoparticles and Its Dependency on Ce ^3+^ Surface Area Concentration. Nanoscale.

[37] Baldim V., Yadav N., Bia N., Graillot A., Loubat C., Singh S., Karakoti A.S., Berret J.-F. (2020). Polymer-Coated Cerium Oxide Nanoparticles as Oxidoreductase-like Catalysts. ACS Appl. Mater. Interfaces.

[38] Goujon G., Baldim V., Roques C., Bia N., Seguin J., Palmier B., Graillot A., Loubat C., Mignet N., Margaill I. (2021). Antioxidant Activity and Toxicity Study of Cerium Oxide Nanoparticles Stabilized with Innovative Functional Copolymers. Adv. Health Mater..

[39] Berret J.-F., Graillot A. (2022). Versatile Coating Platform for Metal Oxide Nanoparticles: Applications to Materials and Biological Science. Langmuir.

[40] Asati A., Santra S., Kaittanis C., Perez J.M. (2010). Surface-Charge-Dependent Cell Localization and Cytotoxicity of Cerium Oxide Nanoparticles. ACS Nano.

[41] Asati A., Santra S., Kaittanis C., Nath S., Perez J.M. (2009). Oxidase-Like Activity of Polymer-Coated Cerium Oxide Nanoparticles. Angew. Chem. Int. Ed..

[42] Lord M.S., Tsoi B., Gunawan C., Teoh W.Y., Amal R., Whitelock J.M. (2013). Anti-Angiogenic Activity of Heparin Functionalised Cerium Oxide Nanoparticles. Biomaterials.

[43] Pramanik N., De T., Sharma P., Alakesh A., Jagirdar S.K., Rangarajan A., Jhunjhunwala S. (2022). Surface-Coated Cerium Nanoparticles to Improve Chemotherapeutic Delivery to Tumor Cells. ACS Omega.

[44] Ristori S., Grillo I., Lusa S., Thamm J., Valentino G., Campani V., Caraglia M., Steiniger F., Luciani P., de Rosa G. (2018). Structural Characterization of Self-Assembling Hybrid Nanoparticles for Bisphosphonate Delivery in Tumors. Mol. Pharm..

[45] Nele V., Holme M.N., Kauscher U., Thomas M.R., Doutch J.J., Stevens M.M. (2019). Effect of Formulation Method, Lipid Composition, and PEGylation on Vesicle Lamellarity: A Small-Angle Neutron Scattering Study. Langmuir.

[46] Battaglini M., Tapeinos C., Cavaliere I., Marino A., Ancona A., Garino N., Cauda V., Palazon F., Debellis D., Ciofani G. (2019). Design, Fabrication, and In Vitro Evaluation of Nanoceria-Loaded Nanostructured Lipid Carriers for the Treatment of Neurological Diseases. ACS Biomater. Sci. Eng..

[47] Cha B.G., Jeong H.-G., Kang D.-W., Nam M.-J., Kim C.K., Kim D.Y., Choi I.-Y., Ki S.K., Kim S.I., Han J.h. (2018). Customized Lipid-Coated Magnetic Mesoporous Silica Nanoparticle Doped with Ceria Nanoparticles for Theragnosis of Intracerebral Hemorrhage. Nano Res..

[48] Turovsky E.A., Varlamova E.G. (2021). Mechanism of Ca^2+-^Dependent Pro-Apoptotic Action of Selenium Nanoparticles, Mediated by Activation of Cx43 Hemichannels. Biology.

[49] Kim S.H., Ryan T.A. (2009). Synaptic Vesicle Recycling at CNS Synapses without AP-2. J. Neurosci..

